# Rapid genomic and transcriptomic alterations induced by wide hybridization: *Chrysanthemum nankingense* × *Tanacetum vulgare* and *C. crassum* × *Crossostephium chinense* (Asteraceae)

**DOI:** 10.1186/1471-2164-14-902

**Published:** 2013-12-18

**Authors:** Haibin Wang, Jiafu Jiang, Sumei Chen, Weimin Fang, Zhiyong Guan, Yuan Liao, Fadi Chen

**Affiliations:** 1 College of Horticulture, Nanjing Agricultural University, No. 1 Weigang, Nanjing 210095, Jiangsu Province, China; 2 Jiangsu Province Engineering Lab for Modern Facility Agriculture Technology & Equipment, No. 1 Weigang, Nanjing 210095, Jiangsu Province, China

**Keywords:** Genome evolution, Gene transcripts, Cytosine methylation, Wide hybridization, Asteraceae

## Abstract

**Background:**

Hybridization is a major driver of evolution in plants. In a number of plant species, the process of hybridization has been revealed to be accompanied by wide-ranging genetic and epigenetic alterations, some of which have consequences on gene transcripts. The Asteraceae family includes a number of polyploid species, and wide crossing is seen as a viable means of genetically improving ornamental species such as *Chrysanthemum* spp. However, the consequences of hybridization in this taxon have yet to be characterized.

**Results:**

Amplified fragment length polymorphism (AFLP), methylation sensitive amplification polymorphism (MSAP) and cDNA-AFLP profiling of the two intergeneric hybrids *C. nankingense* × *Tanacetum vulgare* and *C. crassum* × *Crossostephium chinense* were employed to characterize, respectively, the genomic, epigenomic and transcriptomic changes induced by the hybridization event. The hybrids’ AFLP profiles included both the loss of specific parental fragments and the gain of fragments not present in either parent’s profile. About 10% of the paternal fragments were not inherited by the hybrid, while the corresponding rate for the maternal parent fragments was around 4–5%. The novel fragments detected may have arisen either due to heterozygosity in one or other parent, or as a result of a deletion event following the hybridization. Around one half of the cDNA-AFLP fragments were common to both parents, about 30% were specific to the female parent, and somewhat under 20% specific to the male parent; the remainder (2.9-4.7%) of the hybrids’ fragments were not present in either parent’s profile. The MSAP fingerprinting demonstrated that the hybridization event also reduced the amount of global cytosine methylation, since > 50% of the parental fragments were methylated, while the corresponding frequencies for the two hybrids were 48.5% and 50.4%.

**Conclusions:**

Combining two different Asteraceae genomes via hybridization clearly induced a range of genomic and epigenomic alterations, some of which had an effect on the transcriptome. The rapid genomic and transcriptomic alterations induced by hybridization may accelerate the evolutionary process among progenies.

## Background

Hybridization has contributed substantially to the evolution of higher plants, both in the context of extending genetic diversity and in enhancing adaptive speciation [1-3]. At least 70% of angiosperm species are polyploid, of which the majorities are allo- rather than autopolyploid [4]. Detailed analysis of the genome of many species held to be diploid has revealed that many of these are in fact cryptic polyploids [5], at various stages of decay back to the diploid state [6-10].

Although been debated for more than a century, hybridization is considered to be a potent evolutionary force of genetic variation and functional novelty and occurs frequently in flower plant [11,12]. The allopolyploid state often offers several adaptive advantages over the diploid state. Adaptive advantages include the acquisition of novel gene combinations which can in some cases promote heterosis [13], the duplication of gene functions which can provide an element of buffering, and the potential to evolve novel functionality which were predicted by McClintock as “genomic shock” [1,14]. Hybridization appears to often be accompanied by changes to both genomic sequences, to the epigenome and to the pattern of gene transcripts [15-22]. Some of the latter have been revealed to have been induced by epigenetic, rather than by genetic changes, in particular as a consequence of altered profiles of cytosine methylation which is one of the major and immediate epigenetic responses of the plant genome to hybridization and also play an important role in the regulation of gene transcripts [23-25].

In plant breeding and domestication process, hybridization is a powerful method to import excellent genes and exquisite traits into hybrids (either caused by additive or non-additive effects), which results in the phenotypic superiority of a hybrid over its parents with respect to traits such as greater biomass, speed of development and yield [26,27]. Compared with interspecific hybridization, intergeneric hybridization is more difficult to succeed, and the overall results have not resolved the controversy as to whether intergeneric hybrids have undergone rapid and directed changes in genome change in their evolutionary history [28-30]. Furthermore, the proportion and categories of DNA or cDNA sequences affected by the species differ in various families. Hence, to promote a better understanding of the success of plants, further independent wide hybridization events need to be analyzed in future studies.

Asteraceae is a large group of angiosperms distributed all over the world includes ploidy states ranging from diploid (eg. *C. nankingense*) to decaploid (eg. *C. crassum*) which is generally considered to be an advanced subjects and at the forefront of the evolution [31-33]. Despite numerous studies showed valuable information about rapid genetic and epigenetic changes in many other plants, as a large species group, little is known about these changes in *Chrysanthemum* even in Asteraceae [34,35]. In the early studies, intergeneric hybrids have been successfully created using a wide range of parental materials and some of these hybrids have proven to make highly vigorous plants [36,37]. Here, DNA-AFLP and MSAP fingerprinting were applied to characterize induced changes in the genome and epigenome, and cDNA-AFLP were used to detect changes to the transcriptome in newly synthesized *C. nankingense* × *Tanacetum vulgare* and *C. crassum* × *Crossostephium chinense* hybrids.

## Methods

### Plant materials

**Figure 1:**
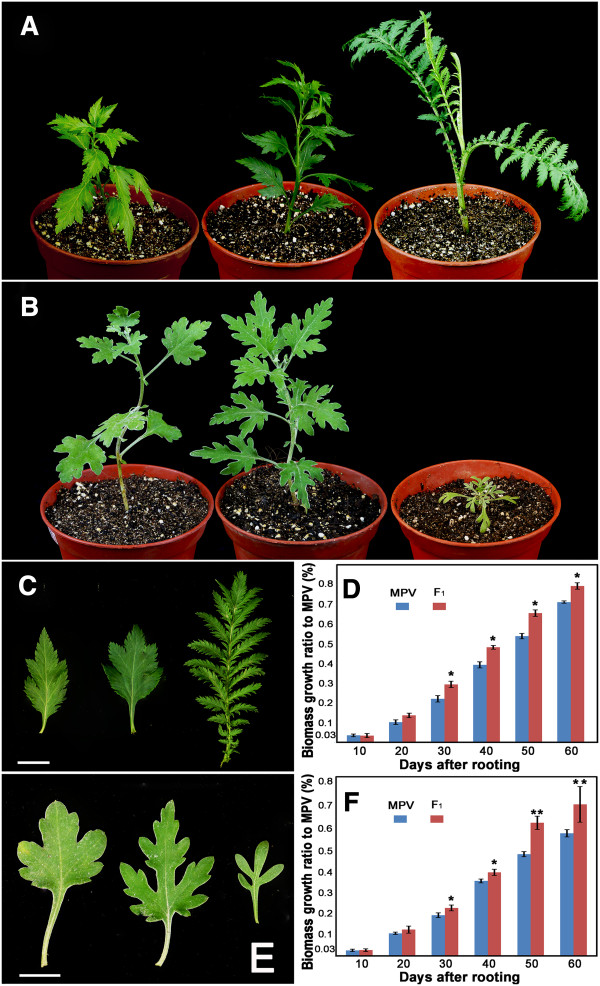
**The phenotype of materials.** The phenotype of **(A)***C. nankingense* (left), *T. vulgare* (right) and their hybrid (center), **(B)***C. crassum* (left), *Cr. chinense* (right) and their hybrid (center).** (C)** Leaf morphology of *C. nankingense* (left), *T. vulgare* (right) and their hybrid (center). Bars: 1 cm. **(E)** Leaf morphology of *C. crassum* (left), *Cr. chinense* (right) and their hybrid (center). Bars: 1 cm. Values shown are the mean and SE of biomass growth ratio to MPV of **(D)** the *C. nankingense* × *T. vulgare* hybrid and **(F)** the *C. crassum* × *Cr. chinense* hybrid, measured ten, 20, 30, 40, 50 and 60 days after rooting. n: number of plants. *P < 0.05; **P < 0.01.

The relevant accessions of *C. nankingense*, *T. vulgare* and their F_1_ hybrid (Figure 1A), and of *C. crassum*, *Cr. chinense* and their F_1_ hybrid (Figure 1B) were obtained from the Chrysanthemum Germplasm Resource Preserving Centre, Nanjing Agricultural University, China (32°05′N, 118°8′E, 58 m altitude). All plants were propagated by cuttings; the medium contained a 2:2:1(v/v) mixture of perlite, vermiculite and leaf mould, respectively. Rooted seedlings were grown in a greenhouse under conditions held at 22°C during the day and at a minimum of 15°C during the night. The relative humidity varied from 70 to 75% (m/m), and no artificial light was given. The experiment included three biological replications.

Intergeneric cross was performed at 9:00–10:00 am on a sunny day, the bisexual tubular florets of female were removed and the inflorescences were enclosed within a paper bag. After two to three days, fresh pollen of the male donor was brushed onto the pistil when the stigmas first became visible and re-bagged. The F_1_ hybrids were obtained via ovule rescue at 10–15 days after pollination [36,37].

### Nucleic acid extraction and cDNA synthesis

Genomic DNA was extracted from fully expanded third and the fourth leaves collected from three biological replication per entry using a CTAB-based method [38], followed by a pectinase and cellulase treatment and the application of a Nuclei Isolation Kit (Solarbio, China). Total RNA was isolated from a similar set of leaves using the TRIzol reagent (Takara, Japan), based on the manufacturer’s protocol. Prior to its reverse transcription, the total RNA preparation was digested for 30 min at 37°C with RNase-free DNase I (Takara, EC 3.1.4.5) to remove any contaminating genomic DNA. The first cDNA strand was synthesized from a 300 ng RNA based on random priming and SuperScript III Reverse Transcriptase (Takara, EC 2.7.7.49). The second strand was then synthesized by the addition of 10 U DNA polymerase I (Takara, EC 2.7.7.7) and 5 U RNase H (Takara, EC 3.1.26.4) [39], and purified by extraction in phenol: chloroform: isoamyl alcohol (25:24:1, v/v) followed by ethanol precipitation. The purified products were each dissolved in 50 μL ddH_2_O.

### DNA/cDNA fingerprinting

The AFLP protocol used to profile the genomic DNA and cDNA was a minor modification of the one described by Vos et al. [40]. The first PCR amplification performed with the AFLP ligation and pre-selective amplification module from PE Biosystems. After diluted in a ratio of 1:30 with ddH_2_O, the PCR product was used as templates for the selective amplification with three selective bases. The selective amplification reactions were based on the nine primer combinations *Eco*RI_2/*Mse*I_5 (abbreviated as ‘E2 + M5’), E2 + M6, E3 + M2, E4 + M3, E4 + M8, E6 + M7, E7 + M3, E8 + M3 and E8 + M7; each primer included three selective bases (sequences of adaptors and primers given in Table 1), and the *Eco*RI primers were labeled with 5-FAM. Each PCR was replicated once, and two aliquots of each reaction were electrophoresed independently through denaturing polyacrylamide gels (5% (v/v) Long Ranger; 36 cm in length) for 2 h at 65 W. Only reproducible fragments in the size range 100–500 bp of two replications were recorded as present (1) or absent (0).

**Table 1:** Sequences of adaptors and primers used for pre-amplification and selective amplification in AFLP and MSAP analysis

**Adaptors/primers**	**Sequence (5**′**–3**′**)**
***Mse*** **I adaptor-1**	GACGATGAGTCCTGAG
***Mse*** **I adaptor-2**	TACTCAGGACTCAT
***Eco*** **RI adaptor-1**	CTCGTAGACTGCGTACC
***Eco*** **RI adaptor-2**	AATTGGTACGCAGTCTAC
***Hpa*** **II/ *Msp *** **I adaptor-1**	GATCATGAGTCCTGCT
***Hpa*** **II/ *Msp *** **I adaptor-2**	CGAGCAGGACTCATGA
***Eco*** **RI pre-selective primer**	GACTGCGTACCAATTCA
***Mse*** **I pre-selective primer**	GATGAGTCCTGAGTAAC
***Hpa*** **II/ *Msp *** **I pre-selective primer**	ATCATGAGTCCTGCTCGG
***Eco*** **RI selective primer-2**	GACTGCGTACCAATTCAAG
***Eco*** **RI selective primer-3**	GACTGCGTACCAATTCACA
***Eco*** **RI selective primer-4**	GACTGCGTACCAATTCACT
***Eco*** **RI selective primer-5**	GACTGCGTACCAATTCACC
***Eco*** **RI selective primer-6**	GACTGCGTACCAATTCACG
***Eco*** **RI selective primer-7**	GACTGCGTACCAATTCAGC
***Eco*** **RI selective primer-8**	GACTGCGTACCAATTCAGG
***Mse*** **I selective primer-2**	GATGAGTCCTGAGTAACAC
***Mse*** **I selective primer-3**	GATGAGTCCTGAGTAACAG
***Mse*** **I selective primer-5**	GATGAGTCCTGAGTAACTA
***Mse*** **I selective primer-6**	GATGAGTCCTGAGTAACTC
***Mse*** **I selective primer-7**	GATGAGTCCTGAGTAACTG
***Mse*** **I selective primer-8**	GATGAGTCCTGAGTAACTT
***Hpa*** **II/ *Msp *** **I selective primer-1**	ATCATGAGTCCTGCTCGGTAA
***Hpa*** **II/ *Msp *** **I selective primer-2**	ATCATGAGTCCTGCTCGGTCC
***Hpa*** **II/ *Msp *** **I selective primer-3**	ATCATGAGTCCTGCTCGGTTC
***Hpa*** **II/ *Msp *** **I selective primer-6**	ATCATGAGTCCTGCTCGGTAG
***Hpa*** **II/ *Msp *** **I selective primer-7**	ATCATGAGTCCTGCTCGGTTG
***Hpa*** **II/ *Msp *** **I selective primer-8**	ATCATGAGTCCTGCTCGGTCA

Differential methylation of the genomic DNA was analyzed by the AFLP-based MSAP technique, based on the isoschizomeric pair *Hpa*II (New England Biolabs, China, EC 3.1.23.24) and *Msp*I (NEB, EC 3.1.23.24), in combination with *Eco*RI (NEB, EC 3.1.23.13). Methylation polymorphisms at 5′-CCGG-3′ tetranucleotide sites generate differences between the *Eco*RI-*Hpa*II (H lanes) and *Eco*RI-*Msp*I (M lanes) profiles [41,42]. About 500 ng of each cDNA was digested with either 10 U *Eco*RI and 20 U *Hpa*II or 10 U *Eco*RI and 10 U *Msp*Iat 37°C for 12 h. The digested fragments were ligated to 5 pmol *Eco*RI adaptor and 50 pmol *Hpa*II–*Msp*I adaptor by incubation with 4 U T4 DNA polymerase (NEB, EC 2.7.7.7) at 16°C for 4 h. Amplicons derived from a pre-selective amplification based on *Eco*RI pre-selective primer and *Hpa*II/*Msp*I pre-selective primer formed the template for a subsequent selective amplification.Selective amplification reaction was based on one of nine primer combinations (E2 + HM6, E4 + HM3, E4 + HM7, E4 + HM8, E6 + HM6, E6 + HM8, E7 + HM1, E8 + HM2 and E8 + HM8; adaptor and primer sequences listed in Table 1). Reaction products were electrophoresed in the same way as were the AFLP products. Electrophoresis profiles were used to derive a difference between the mid-parental value and the hybrids, according to the formula,

$$\begin{array}{l} p=\frac{y1+y2}{n1+n2};q=1-p;\delta_{p1-p2}=\sqrt{\mathrm{pq}\left(\frac{1}{n1}+\frac{1}{n2}\right)};\\ U=\frac{p1-p2}{\delta_{p1-p2}} \end{array}$$

In which *n*1 represented the total sites of the mid-parent values, *n*2 the number of fragments in their hybrid, y1 the total DNA methylation sites, hemimethylation sites or fully methylation sites of the mid-parent values, y2 represents the total DNA methylation sites, hemimethylation sites, or fully methylation sites of a hybrid, *p*1 the percentage of total methylation sites, hemimethylation sites or fully methylation sites for the mid-parent values and *p*2 the percentage of total methylation sites, hemimethylation sites or fully methylation sites for a hybrid.

## Results and discussion

### Alterations in the genome sequence of the newly synthesized allopolyploids

**Figure 2:**
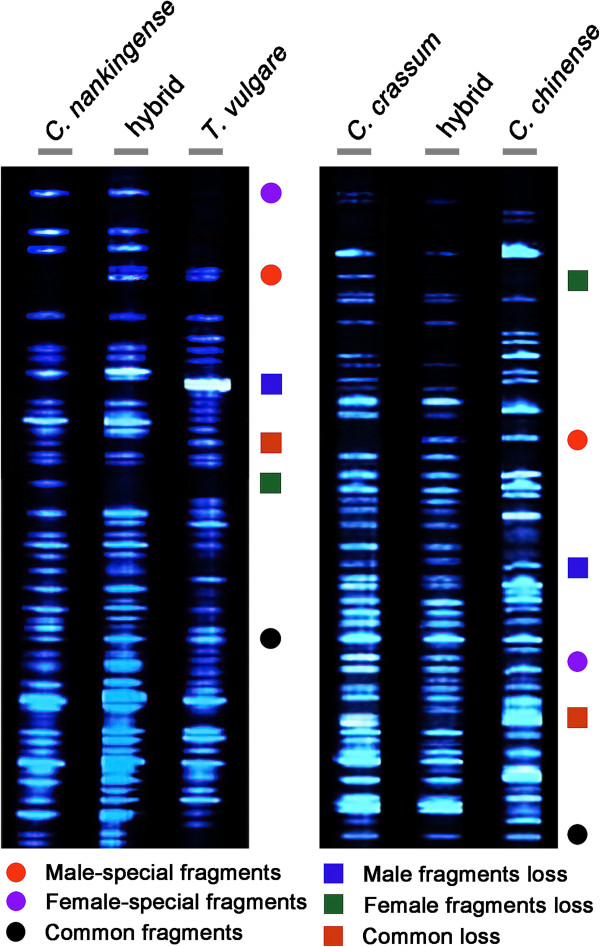
Typical AFLP profiles of the hybrids and their parents.

As all hybrids were obtained from inbred parental lines and no parental fragment disappearance was not caused by chromosome elimination in the previously published studies [36,37], every independently made F_1_ hybrid had the same phenotypic and band patterns, one would not expect to observe much change according to conventional theory. In fact, hybrids fragments were nearly all inherited from their maternal parent. For example, of the total of 429 AFLP fragments detected in the *C. nankingense* × *T. vulgare* hybrid, all but four (i.e., 99.1% of all AFLP fragments) were present in one or both of the parents. The same frequency applied for the *C. crassum* × *Cr. chinense* hybrid (445/449 fragments; Figure 2, Table 2). In addition, about > 40% common fragments, subjects heterozygous for genetic polymorphism compared to parents show a significant consequence of hybridization effect, herein refers to > 22% male-special fragments and > 34% female-special fragments (Figure 2 and Table 2). A relatively high frequency of maternal fragments are also identified for some RFLP analyses of *Phaseolus* hybrids [43,44], suggesting that differential transmission of gene loci in the present study may be not a random trait derived from hybridization.

**Table 2:** Fragments type in two independent DNA-AFLP analyses

**Fragments type**	***C. nankingense*** **×** ***T. vulgare***		***C. crassum*** **×** ***Cr. chinense***	
	**Number**	**Percentage**	**Number**	**Percentage**
**Common fragments**	184	42.9%	180	40.1%
**Female-special fragments**	146	34.0%	162	36.1%
**Male-special fragments**	95	22.1%	103	22.9%
**Novel fragments**	4	0.9%	4	0.9%
**Total fragments**	429	100%	449	100%

Besides the additives from the contributing parents, the nascent F_1_ hybrids often show subtle fragment variations. Two major types of genetic changes are recognized in the F_1_ hybrid plant: loss of parental fragments (Figure 2 and Table 3) and gain of novel fragments (Table 3). Both hybrids failed to inherit a number of parental fragments; specifically, around 10% of the paternal fragments were not present in the hybrid profiles (35/321 and 33/325), while the equivalent rate of loss from the maternal parents was 5.1% (18/355) for one hybrid, and 4.4% (16/367) for the other. The rates are all somewhat higher than what has been reported in newly synthesized hybrids such as wheat [29]. Although, loss events may reflect some residual heterozygosity in the parents, the greatest possible or the most of loss fragment is more likely to represent spontaneous deletions occurred as a manifestation of genomic shock in the process of hybridization [1,4,8,11,45]. Interestingly, compared to loss events, the novel fragments were calculated as only 0.9% (Table 3). The non-parental AFLP fragments in the DNA-AFLP analysis might be the result of allelic variation present in one of the parental species or also a spontaneous deletion that occurred in the F_1_ hybrid.

**Table 3:** **DNA-AFLP fragments loss type in F**_**1 **_**hybrids and their corresponding parents**

**Fragments type**	***C. nankingense*** **×** ***T. vulgare***				***C. crassum*** **×** ***Cr. chinense***			
	**Number**	**Total number**			**Number**	**Total number**		
		**Female**	**Hybrid**	**Male**		**Female**	**Hybrid**	**Male**
**Female fragments loss**	18	355	**-**	-	16	367	**-**	**-**
**Male fragments loss**	35	**-**	**-**	321	33	**-**	**-**	325
**Common loss**	7	**-**	**-**	-	9	**-**	**-**	**-**
**Novel fragments**	4	**-**	429	-	4	**-**	449	**-**

A variety of analytical platforms has been exploited to show that *de novo* synthesized hybrids undergo massive genetic (chromosomal rearrangements, DNA sequence elimination) and epigenetic adjustment [3,17,46]. Rearrangements and deletions both have the potential to generate non-parental AFLP fragments in the hybrid’s genomic DNA, if rearrangements and deletions affect restriction sites targeted by the procedure. Current consensus view is that the process of polyploidization is accompanied by the elimination of both low copy and/or non-coding DNA sequence [18,47-50]. In synthetic wheat hybrids, deletion events have been proposed to be a major driver of the observed genomic changes [18], and an essentially similar conclusion was arrived at in *Cucumis*[51], *Brassica*[52] and *Tragopogon*[19]. Extensive loss of parental AFLP fragments from the hybrid’s genome was a feature of both the *C. nankingense* × *T. vulgare* and the *C. crassum* × *Cr. chinense* combinations. The deletion events were likely to have occurred very early in the process of hybrid zygote formation.

### Alterations in the epigenome of the newly synthesized allopolyploids

**Figure 3:**
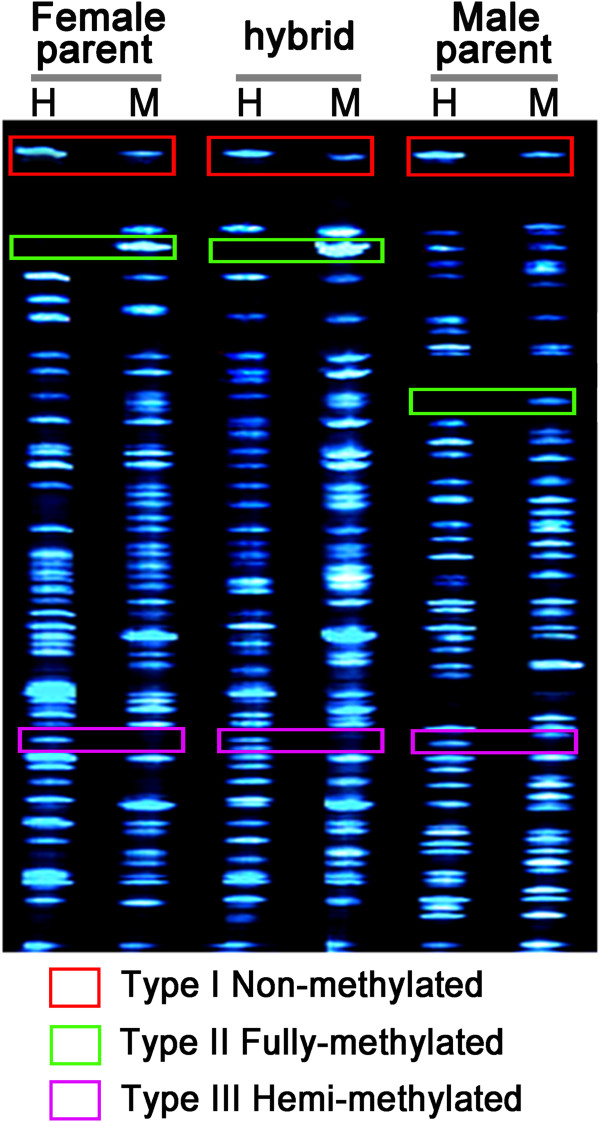
Typical cDNA-AFLP profiles of the hybrids and their parents.

Variation in the epigenome was explored via the MSAP technique, as illustrated in Figure 3. Type I (non-methylated) fragments were shared by the H and M lanes, type II (fully methylated) were only detected in M lanes, while type III (hemi-methylated) appeared only in the H lanes. The nine primer combinations employed generated 274 type I, 178 type II and 132 type III fragments in *C. nankingense*, and respectively 275, 164 and 133 in *T. vulgare*; the equivalent numbers of fragments in *C. crassum* were 261, 171 and 153, and in *Cr. chinense* 268, 162 and 142 (Table 4). Surprisingly, the global DNA methylation concentration in the diploids (51.9-53.1%) was not much lower than that in the decaploid (55.4%), as the expectation is that higher concentrations of ploidy are generally associated with more extensive DNA methylation.

**Table 4:** **Levels of cytosine methylation in F**_**1 **_**hybrids and their corresponding parents**

**Plant lines**	**Total sites**	**Non-methylated**	**Methylated**		
		**Type I**	**Total**	**Type II**	**Type III**
***C. nankingense***	584	274 (46.9%)	310 (53.1%)	178 (30.5%)	132 (22.6%)
***T. vulgare***	572	275 (48.1%)	297 (51.9%)	164 (28.7%)	133 (23.3%)
**Mid-parental value**	100%	47.5%	52.5%	29.6%	22.9%
**Hybrid**	567	292 (51.5%)	275 (48.5%)	148 (26.1%)	127 (22.4%)
***C. crassum***	585	261 (44.6%)	324 (55.4%)	171 (29.2%)	153 (26.2%)
***Cr. chinense***	572	268 (46.9%)	304 (53.1%)	162 (28.3%)	142 (24.8%)
**Mid-parental value**	100%	45.7%	54.3%	28.8%	25.5%
**Hybrid**	573	284 (49.6%)	289 (50.4%)	149 (26.0%)	140 (24.4%)

Meanwhile, polymorphic fragments were also scored as methylation changes between hybrids and parents. The amount of cytosine methylation in the hybrids were 48.5% for *C. nankingense* (53.1%) × *T. vulgare* (51.9%; *U* = 1.36, *U*_0.05_ = 1.96) and 50.4% for *C. crassum* (55.4%) × *Cr. chinense* (53.1%; *U* = 1.31, *U*_0.05_ = 1.96; Table 4). With respect to fully methylated sites, the hybrids displayed lower *U* values than predicted on the basis of mid-parent value (*C. nankingense* × *T. vulgare*: *U* = 1.31, *C. crassum* × *Cr. chinense*: *U* = 1.06). With respect to the hemi-methylated sites, the respective *U* values were only 0.21 and 0.47.

Present results suggested that the adjustments of DNA methylation patterns occurred widely at various genomic sites in each of the hybrid plants (Table 4). Combining two divergent genomes of distinct parental species in a new plant must generate the strong “shock”, may disrupt intrinsic regulatory and developmental harmonies, possibly cause a myriad of incompatibilities at many layers, which is particularly important in plant evolution [3,14,27,53]. The occurrence and extent of methylation variation are dependent on genetic context of the hybrid. Nonetheless, the relative total frequencies of variation between the hybrids for a given combination are remarkably similar according to the present results. Thus, the similarity between the MSAP profiles of independent hybrids shows that epigenetic events do not occur stochastically, but rather are pre-determined in some way and might be a rapid process that occurred as early as in the F_1_ hybrid.

### Induced differences in the transcriptome

**Figure 4:**
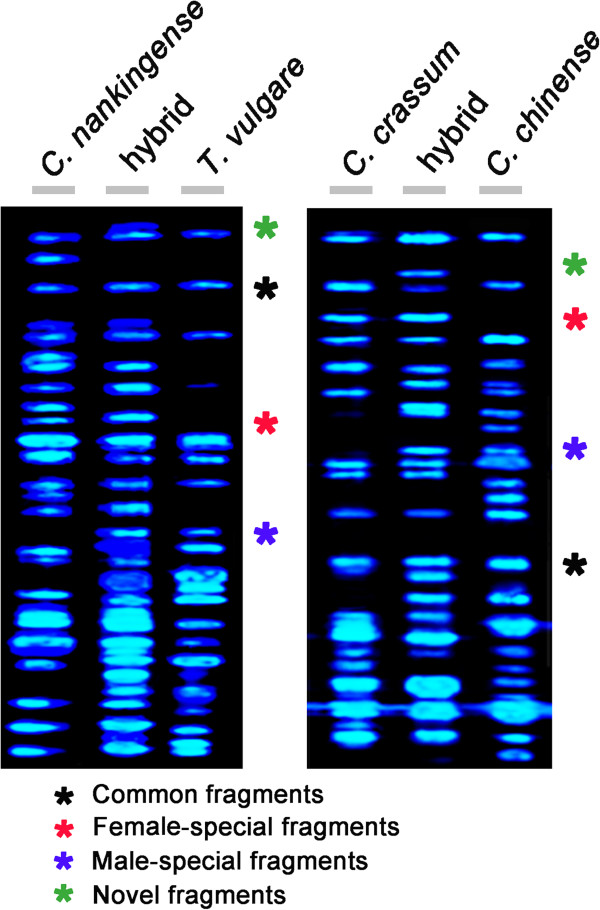
**The various types of fragment generated by MSAP.** Type I fragments are non-methylated, and appeared in both the H and M lanes, type II are fully-methylated, and were only detected in the M lanes, while type III are hemi-methylated, and were only detected in the H lanes.

Both hybrids grow larger and faster than the best of their parents (Figure 1D, F) [36,37]. cDNA-AFLP profiling defined sets of transcript fragments which were either: (a) common to both parents and the hybrid (50.5% of all the fragments in the *C. nankingense* × *T. vulgare* cross and 52.2% in the other cross; (b) fragments which were specific to the female parent and which were inherited by the hybrid (29.6% and 28.6%); (c) fragments which were specific to the male parent and which were inherited by the hybrid (16.9% and 14.5%), and (d) fragments in the hybrid which were not present in either parent (3.0% and 4.7%; Figure 4, Table 5). Meanwhile, there are 8 of *C. nankingense*, 22 of *T. vulgare* and 9 of *C. crassum*, 19 of *Cr. chinense* cDNA fragments missed in cDNA-AFLP detection (Table 6), indicated that transcriptome divergence is reconciled during intergeneric hybridization but weaker than DNA sequences adjustment. Fragments of this sort may reflect the outcome of DNA sequence elimination and genome reorganization, although the possibility of organellar origin gene (in particular those sited in the chloroplast) expression or regulation cannot be excluded, since these cDNA is only transmitted to the hybrid via the female gamete.

**Table 5:** Fragments type in two independent cDNA-AFLP analyses

**Fragments type**	***C. nankingense*** **×** ***T. vulgare***		***C. crassum*** **×** ***Cr. chinense***	
	**Number**	**Percentage**	**Number**	**Percentage**
**Common fragments**	155	50.5%	166	52.2%
**Female-special fragments**	91	29.6%	91	28.6%
**Male-special fragments**	52	16.9%	46	14.5%
**Novel fragments**	9	3.0%	15	4.7%
**Total fragments**	307	100%	318	100%

**Table 6:** **cDNA-AFLP fragments loss type in F**_**1 **_**hybrids and their corresponding parents**

**Fragments type**	***C. nankingense*** **×** ***T. vulgare***				***C. crassum*** **×** ***Cr. chinense***			
	**Number**	**Total number**			**Number**	**Total number**		
		**Female**	**Hybrid**	**Male**		**Female**	**Hybrid**	**Male**
**Female fragments loss**	8	259	**-**	-	9	271	**-**	**-**
**Male fragments loss**	22	**-**	**-**	234	19	**-**	**-**	236
**Common loss**	5	**-**	**-**	-	7	**-**	**-**	**-**
**Novel fragments**	9	**-**	307	-	15	**-**	318	**-**

Transcriptomic studies of hybridization in plants have revealed that patterns of gene transcripts likely have a profound effect in a hybrid context [15]. In spite of intensive study for approximately a century, the molecular basis of heterosis remains unclear. Genome-wide transcriptomic alterations correlates with the expression divergence between the parents have been observed in the hybrid [13,26,54]. Expression profiles in hybrids formed from very wide crosses have repeatedly been revealed to be non-additive, which provides a possible molecular lead in explaining heterosis [27] and phenotypic variation in the hybrid progeny [24]. An admitted suggestion holds that epigenetic mechanisms are important for regulating the relative abundance of gene transcripts [25,55]. Genomic shock can disrupt a number of regulatory and developmental processes, particularly via changes to the epigenome given that hypermethylation is associated with gene silencing, whereas hypomethylation is often associated with gene activity [56]. The MSAP analysis suggested that DNA methylation was at a lower degree in the hybrids than in their corresponding parents, a finding which could explain the origin of at least some of the non-parental cDNA-AFLP fragments present in the hybrids [57,58]. Elucidating the ways in which altered DNA methylation patterns, either at the whole genomic level or at specific sites can affect genome stability during a hybridization event will require substantial additional investigation [59].

## Conclusion

In conclusion, large scale genomic, epigenomic and transcriptomic changes accompanied the process of hybridization in the crosses *C. nankingense* × *T. vulgare* and *C. crassum* × *Cr. chinense*. The forced union of two distinct genomes induced many changes to both the genome and the transcriptome. The former changes were largely brought about by the elimination of DNA, while the latter reflected in addition the effect of altered amount of cytosine methylation. Together, these rapid changes could drive the evolutionary process of the freshly formed intergeneric hybrids.

## Competing interests

The authors have no competing interests to declare.

## Authors’ contributions

Conceived and designed the experiments: HW FC JJ SC. Performed the experiments: HW FC JJ. Analyzed the data: HW JW. Contributed reagents/materials/analysis tools: WF ZG. Wrote the paper: HW JJ SC. All authors read and approved the final manuscript.
